# Vertical Transmission of Gut Dysbiosis From Mothers With Gestational Diabetes to Infants

**DOI:** 10.1111/1753-0407.70148

**Published:** 2025-10-01

**Authors:** Jia Ming Low, Abhishek Gupta, Rachel Toh, Su Lin Lim, Shiao‐Yng Chan, Sanjay Swarup, Le Ye Lee

**Affiliations:** ^1^ Department of Neonatology, Khoo Teck Puat‐National University Children Medical Institute, National University Hospital National University Health System Singapore Singapore; ^2^ Department of Paediatrics, Yong Loo Lin School of Medicine National University of Singapore Singapore Singapore; ^3^ Singapore Centre for Environmental Life Sciences Engineering (SCELSE) National University of Singapore Singapore Singapore; ^4^ Department of Paediatrics, Khoo Teck Puat‐National University Children Medical Institute, National University Hospital National University Health System Singapore Singapore; ^5^ Office of Allied Health & Pharmacy, National University Hospital National University Health System Singapore Singapore; ^6^ Department of Obstetrics and Gynaecology, National University Hospital National University Health System Singapore Singapore; ^7^ Department of Biological Sciences National University of Singapore Singapore Singapore; ^8^ NUS Environmental Research Institute (NERI) Singapore Singapore; ^9^ Singapore Paediatrics Care for Kids (SPARK) Mount Alvernia Hospital Singapore Singapore

**Keywords:** biosignatures, early life gut microbiome, gestational diabetes mellitus (GDM), mother‐infant dyad, trans‐generational or vertical transfer

## Abstract

**Background:**

Vertical transmission of microbes from a mother's gut to their offspring plays a crucial role in the genesis of the early life gut microbiome. Gestational Diabetes Mellitus (GDM) is the commonest metabolic disorder during pregnancy, which has adverse short‐ and long‐term effects on both maternal and infant health. We aimed to capture the GDM‐associated biosignatures in infants' gut microbiome from birth to the first 6 weeks of life.

**Methods:**

53 GDM mother‐infant dyads and 16 healthy mother‐infant dyads were recruited. We performed targeted 16S rRNA gene amplicon sequencing on stool samples. Various statistical analyses were performed to understand the changes in the microbiome profile of infants and identify GDM‐associated bacterial biomarkers in mothers and their transfer to infants.

**Results:**

GDM altered the gut microbiome of pregnant women as compared to healthy counterparts (PERMANOVA, *p*.adjusted < 0.05), with predominance of bacterial members associated with insulin resistance, proinflammatory conditions, and other metabolic processes. Infants born to GDM mothers have distinctive early life microbiome (meconium and six weeks stools) compared to infants born to control mothers (PERMANOVA, *p*.adjusted < 0.05). We also identified the presence of various GDM‐associated microbial signatures such as *Blautia* and *Collinsella* in both meconium and one‐month‐old stool samples of infants born to GDM mothers.

**Conclusion:**

This study provides a better understanding of the impact of GDM on the seeding of a specific set of microbes during the early life colonization event in the infant gut that increases the risk of inflammatory and metabolic diseases in the future.

## Graphical abstract

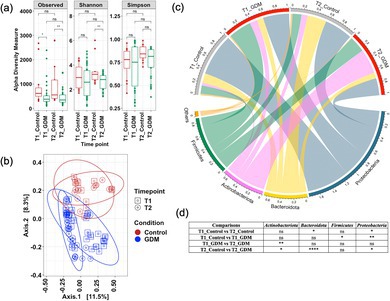

**Summary**

- Infants born to mothers with Gestational Diabetes Mellitus (GDM) had a distinctive early life microbiome (meconium and six weeks old stools) compared to infants born to mothers without GDM.
- There is a transfer and retention of GDM‐associated bacterial taxa such as *Collinsella*, *Blautia*, and *Ruminococcus* in infants born to mothers with GDM.
- Alterations in the maternal microbiome due to GDM can influence the infant's gut microbiome. This highlights the potential for microbiota‐based interventions to enhance maternal metabolic health.

**Abbreviations**
ASVs: amplicon sequence variants
FEAST: fast expectation–maximization microbial source tracking
GDM: gestational diabetes mellitus
OGTT: oral glucose tolerance testing
PCoA: principal coordinate analysis
PERMANOVA: permutational multivariate analysis of variance

## 1 Introduction

Early life events from conception to infancy can potentially impact lifelong consequences in human health [1]. One of these early life events is the vertical transmission transfer of microbes from mother to their offspring, which plays a significant role in the genesis of the early life gut microbiome [2]. This transfer of gut microbiome from mothers to neonates is dependent on various factors including the mode of delivery, antibiotic usage, type of milk feeds given, gestational age at birth, host genetics, environment, and the health state of the mother [3, 4]. If a mother with an underlying medical pathology has an unhealthy/unbalanced microbiome, this may lead to the transfer of harmful microbes to the infant, which could interfere with the infant's metabolic and immune development in his or her childhood [4]. Therefore, it is conceivable that the early infant gut microbiome could provide insights into the future health condition of the developing infant and also provide a window of opportunity to predict and intervene to prevent potential health complications in the later stages of life.

Gestational Diabetes Mellitus (GDM) is the commonest metabolic phenomenon during pregnancy and has adverse short‐ and long‐term effects on the mother and infant health [5]. Various studies have reported that GDM alters the gut microbiome of pregnant women by enriching the bacterial taxa such as *Collinsella, Blautia, Ruminococcus, Eubacterium halli* group, etc. [6, 7, 8, 9, 10] In addition, the dysbiosis from GDM has been reported on the neonatal gut microbiome at birth [6, 9]. Hence, colonization of the infant gut with such bacterial taxa provides a clue to understand the probability of having metabolic or cardiovascular disease in the near future [11]. However, the trans‐generational transfer of GDM‐associated microbes from mother to offspring in the infant gut microbiome remains unclear [6, 9]. It is critical to understand the assemblage and evolution of the infant gut microbiome after birth and the role of these microbes in the regulation of infant metabolism as the gut microbiome undergoes multiple stages of maturation till its final establishment as the adult gut microbiome [12].

In this prospective cohort study, we performed targeted 16S rRNA gene amplicon sequencing of meconium and stool samples of infants born to GDM and non‐GDM (i.e., control) mothers along with stool samples of pregnant women with and without GDM. The presence or absence of GDM was included to determine the variations of maternal and neonatal microbiome corresponding to the health state of the pregnant women, as well as to illustrate the underlying microbial differentiations in early life up to 6 weeks of life in the infants. In addition, vertical transmission of microbiome as well as GDM‐associated microbial taxa from GDM mothers to their offspring via mother–child pairing was also tracked.

## 2 Methods

### 2.1 Study Design, Subject Recruitment, and Ethical Clearance

For the present prospective cohort pilot study, pregnant women were recruited at the National University Hospital, Singapore (NUH) from 09 April 2019 to 31 December 2021. Pregnant women who were eligible for the study had to fulfill the following strict inclusion criteria: (i) Chinese, Malay, or Indian descent; (ii) aged between 25 and 40 years; (iii) body mass index at oral glucose tolerance testing (OGTT) testing between 20 and 35 kg/m^2^; (iv) no maternal setup for sepsis; (v) not on probiotic supplements or antibiotics for a month; (vi) no other significant maternal co‐morbidities; (vii) planning to deliver via vaginal delivery; and, (viii) compliant to follow up/treatment. Pregnant women with suspected major foetal malformations or chromosomal abnormalities were not approached for recruitment. All recruited pregnant women underwent OGTT between 24 and 28 weeks of gestation based on the Ministry of Health (MOH), Singapore, Clinical Practice Guidelines 1/2014. The diagnosis of GDM was made based on the results of the OGTT. GDM was diagnosed using WHO (World Health Organization) 2013 criteria: fasting plasma glucose ≥ 5.1 mmol/L, or a 1‐h glucose ≥ 10.0 mmol/L, or a 2‐h glucose ≥ 8.5 mmol/L. Recruited pregnant women who met the above criteria and had OGTT results above the threshold were considered as GDM. Recruited pregnant women who met the above criteria and who did not have GDM were the control group. Detailed description on maternal characteristics is published in our previous study [13].

After the recruited women delivered, their newborns were included if they fulfilled the following criteria: (i) born full term (i.e., > 37 weeks gestation); (ii) a birth weight of more than 2.5 kg; and (iii) no major congenital or post‐natal complications.

Ethical clearance was obtained prior to the conduct of this prospective cohort study from the National Healthcare Group Domain Specific Review Board (NHG DSRB), Singapore (reference no.: 2019/00064). Since this was a pilot study, we selected a convenience sample size.

### 2.2 Stool and Meconium Sample Collection, DNA Extraction, and 16S rRNA Gene Sequencing

The stool samples from GDM and control pregnant women were collected between the period of 36–40 gestational weeks of pregnancy (before delivery). Neonatal stool samples were collected at two time points; first passage of stool at birth (i.e., meconium stools henceforth labeled as T1) and stools at 6 weeks of life (henceforth labeled as T2). All stool samples were collected at room temperature using a sterile plastic spoon and placed into a sterile container. The samples were transferred to the laboratory at room temperature and stored at −80°C till DNA extraction. Total DNA was extracted from the stool samples using the CTAB/SDS method. Total DNA was further subjected to amplification of V3‐V4 regions using 341F and 806R primers. The amplified product was sequenced on the NovoSeq 6000 platform with 250X2 bp chemistry at NovogeneAIT Genomics Singapore Pte Ltd. Mothers' data [maternal characteristics and 16S rRNA gene amplicon (V3‐V4 region)] used in the present study was obtained from our previous study [13].

### 2.3 16S rRNA Gene Sequence Analysis and Statistics

The 16S rRNA gene sequences were processed and analyzed using DADA2 package v1.26.0 [14]. Primers and low‐quality bases were removed from the raw reads. Taxonomic assignment was performed on non‐chimeric amplicon sequence variants using SILVA Database (silva _nr‐99_v138.1_train_set.fa.gz). Phyloseq v3.4.2 R package was used for generating both alpha and beta diversity metrics [15]. Pairwise Wilcoxon test was used to compare the alpha diversity metrics and relative abundance of bacterial taxa between categories or groups. Principal coordinate analysis (PCoA) was performed with Bray‐Curtis's dissimilarity matrix to understand the difference in the community composition of mother and child. Permutational multivariate analysis of variance (PERMANOVA) with adjusted *p*‐value (FDR corrected) was performed using the Bray Curtis dissimilarity matrix to assess the difference in beta diversity using pairwise adonis function. R packages such as circlize [16] was used to generate the circus plot of major phylum, while ggalluvial [17] was used to generate the alluvial flow diagram of major bacterial genera. DeSeq2 [18] analysis was performed to identify the differentially enriched or depleted ASVs between the groups (T1: meconium of infants born to GDM mothers versus control infants born to non‐GDM mothers; T2 stools of infants born to GDM mothers versus control infants born to non‐GDM mothers, and stools of both control and GDM mothers who had delivered via vaginal delivery) using the Wald test and an adjusted *p*‐value of < 0.01. The functional prediction of stool microbiome of infants was performed using the phylogenetic investigation of communities by reconstruction of unobserved states (PICRUSt2) [19], followed by identification of statistically significant differentially abundant functional KEGG categories/metabolisms between T1 and T2 stool samples of infant born to GDM and control mothers using STAMP software [20] implemented with Welch's t‐test; *p*‐values were corrected using the Benjamini‐Hochberg FDR method.

Core microbiome (prevalence > 90%) of mother samples who delivered vaginally belonging to GDM and control categories were identified separately using the microbiome package [21]. These core microbial taxa were then identified in infants and a heatmap with hierarchical clustering (Ward's method) was generated using pheatmap package [22]. Similarly, based on DeSeq2 analysis of stool samples associated to GDM mother and control samples, top ASVs enriched in GDM mothers were identified (Log2 fold change was higher than 3.5 with adjusted *p <* 0.001) and heatmap‐based distribution of these ASVs in infants' samples was plotted. Non‐metric multidimensional scaling (NMDS) ordination plot was generated using these GDM‐associated ASVs with Bray‐Curtis's dissimilarity matrix to understand the difference between infants born to GDM and control mothers. The relative contribution of mothers' and meconium microbiome in stool samples of infants (6 weeks old) was estimated using fast expectation–maximization microbial source tracking (FEAST) [23].

Low abundant ASVs (cumulative abundance across the samples below 10) were removed to perform the FEAST analysis; these same datasets were used to identify the vertically transferred ASVs from mother to meconium and meconium to stool manually. Vertical transfer of ASVs was examined through mother‐infant pairing. The number of unique (only present in meconium) and shared ASVs (transferred from mother) was identified in T1 stool samples based on individual mother‐infant pairs. We calculated the percentage of shared ASVs in meconium which were transferred to the T2 stool samples (calculation: number of shared ASVs transferred from T1 to T2 infant stools divided by total number of shared ASVS present in meconium * 100). We then calculated the percentage of transferred ASVs (from T1 to T2 infant stools) out of total ASVs of T2 infant stools (calculation transferred ASVs from T1 infant stools (shared by mother) to T2 infant stools divided by total ASVs of T2 infant stools * 100).

## 3 Results

### 3.1 Subject Recruitment and Samples Collected

**FIGURE 1:**
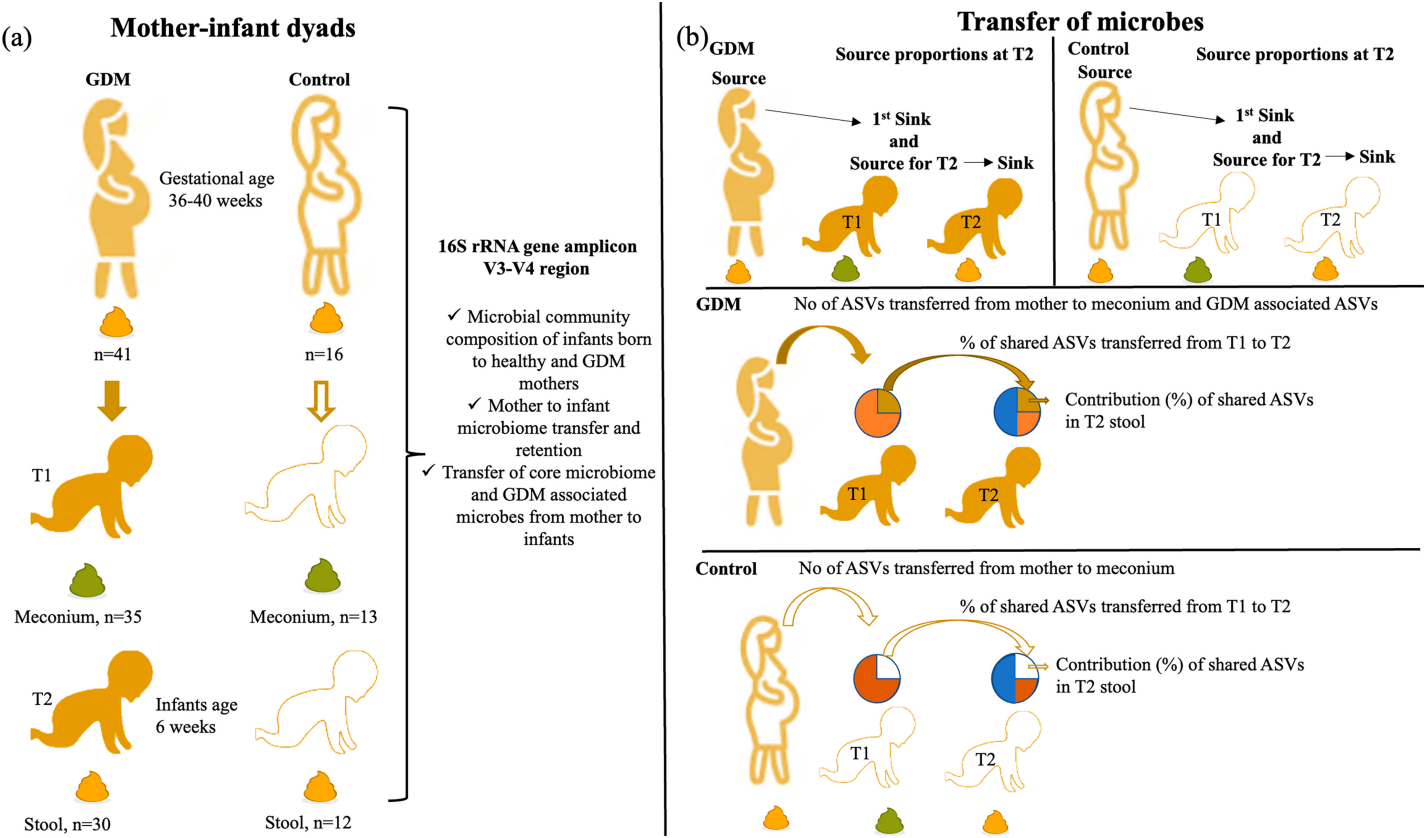
Detailed description of study design and approaches used to decipher the (a) gut microbiome profile and (b) vertical transmission events in mother‐infant dyads. Numbers denote the samples that passed the amplicon sequencing and were used for analysis.

In total, 112 pregnant women were recruited for this cohort study. Of those recruited, 16 belonged to the non‐GD (control) category while 96 were diagnosed with GDM. Forty‐three GDM women were excluded from the analysis as they did not meet the inclusion criteria or dropped out from the study citing personal reasons. As a result, 53 GDM and 16 control mother‐infant dyads were included in the study. Out of the 53 GDM mother‐infant dyads, 43 had all 3 samples collected (i.e., maternal stools, meconium, and six weeks old infant stools), whereas 10 GDM mother–child dyads only had 2 samples collected (i.e., maternal stools and T1 stools). All 3 samples were collected from the 16 mother‐infant control dyads. The clinical characteristics of GDM and control dyads are listed in Table 1. Microbiome data were successfully generated for 41 GDM and 16 control maternal stools. Additionally, we sequenced meconium (T1: GDM; *n* = 35 and control; *n* = 13) and 6‐week‐old infants' stool samples (T2: GDM; *n* = 30 and control; *n* = 12); these were used in the downstream analysis. The detailed downstream analysis approach is provided in Figure 1a,b.

**TABLE 1:** Demographics of GDM and control (i.e., non‐GDM) mother‐infant dyads.

Characteristics	GDM mothers (*n* = 53)	Control mothers (*n* = 16)
Maternal age (years), mean (SD)	32.8 (3.7)	32.7 (2.9)
Ethnicity, *n* (%)		
Chinese	27 (50.9)	6 (37.5)
Malay	14 (26.4)	5 (31.3)
Indians	12 (22.6)	5 (31.3)
GDM in previous pregnancy, *n* (%)		
Yes	5 (9.4)	0 (0.0)
No	48 (90.6)	16 (100.0)
Pre‐pregnancy BMI (kg/m^2^), mean (SD)	26.4 (4.4)	24.6 (3.7)
Intervention given for GDM, *n* (%)		
Dietary counseling	43 (81.1)	—
Insulin	10 (18.9)	—
Fasting glucose at OGTT, mmol/dL, mean (SD)	4.6 (0.5)	4.3 (0.3)
Glucose level at 2 h for OGTT, mmol/dL, mean (SD)	8.8 (1.0)	6.3 (1.2)
Mode of delivery, *n* (%)		
Normal vaginal delivery	42 (79.2)	16 (100.0)
Caesarean section	10 (18.9)	—
Assisted delivery	1 (1.9)	—
Intrapartum antibiotics, *n* (%)		
Yes	25 (47.2)	—
No	27 (50.9)	16 (100.0)
Not applicable	1 (1.9)	—

Neonatal outcomes	Infants born to GDM mothers (*n* = 52)	Infants born to control mothers (*n* = 16)
Neonatal morbidity, *n* (%)		
Preterm delivery < 37 weeks	3 (5.6)	0 (0.0)
Admission to neonatal care unit	6 (11.5)	0 (0.0)
Neonatal hypoglycaemia < 2.5 mmol/dL	6 (11.5)	0 (0.0)
Macrosomia (birthweight > 4 kg)	3 (5.8)	0 (0.0)
Birth injury	0 (0.0)	0 (0.0)
Gestational age at delivery, weeks, mean (SD)	38.2 (1.6)	38.9 (1.1)
Birth weight, g, mean (SD)	3071 (530)	3058 (349)
Gender, *n* (%)		
Male	29 (55.8)	9 (56.3)
Female	23 (44.2)	7 (43.8)
Type of feeds at T1, *n* (%)		
Exclusive breastfeeding	38 (73.1)	16 (100.0)
Formula feeding	3 (5.8)	0 (0.0)
Combination feeding	11 (21.2)	0 (0.0)
Type of feeds at T2, *n* (%)		
Exclusive breastfeeding	21 (40.4)	10 (62.5)
Formula feeding	0 (0.0)	1 (6.3)
Combination feeding	21 (40.4)	4 (25)
Dropped out	10 (19.2)	1 (6.3)
Antibiotic use in neonatal period, *n* (%)		
Yes	6 (11.5)	0 (0.0)
No	46 (88.5)	16 (100.0)

Abbreviations: BMI, body mass index; GDM, gestational diabetes mellitus; OGTT, oral glucose tolerance test; T1, timepoint 1; T2, timepoint 2.

### 3.2 Microbiome Profile of T1 and T2 Stool Samples From Infant Born to GDM and Control Mothers

**FIGURE 2:**
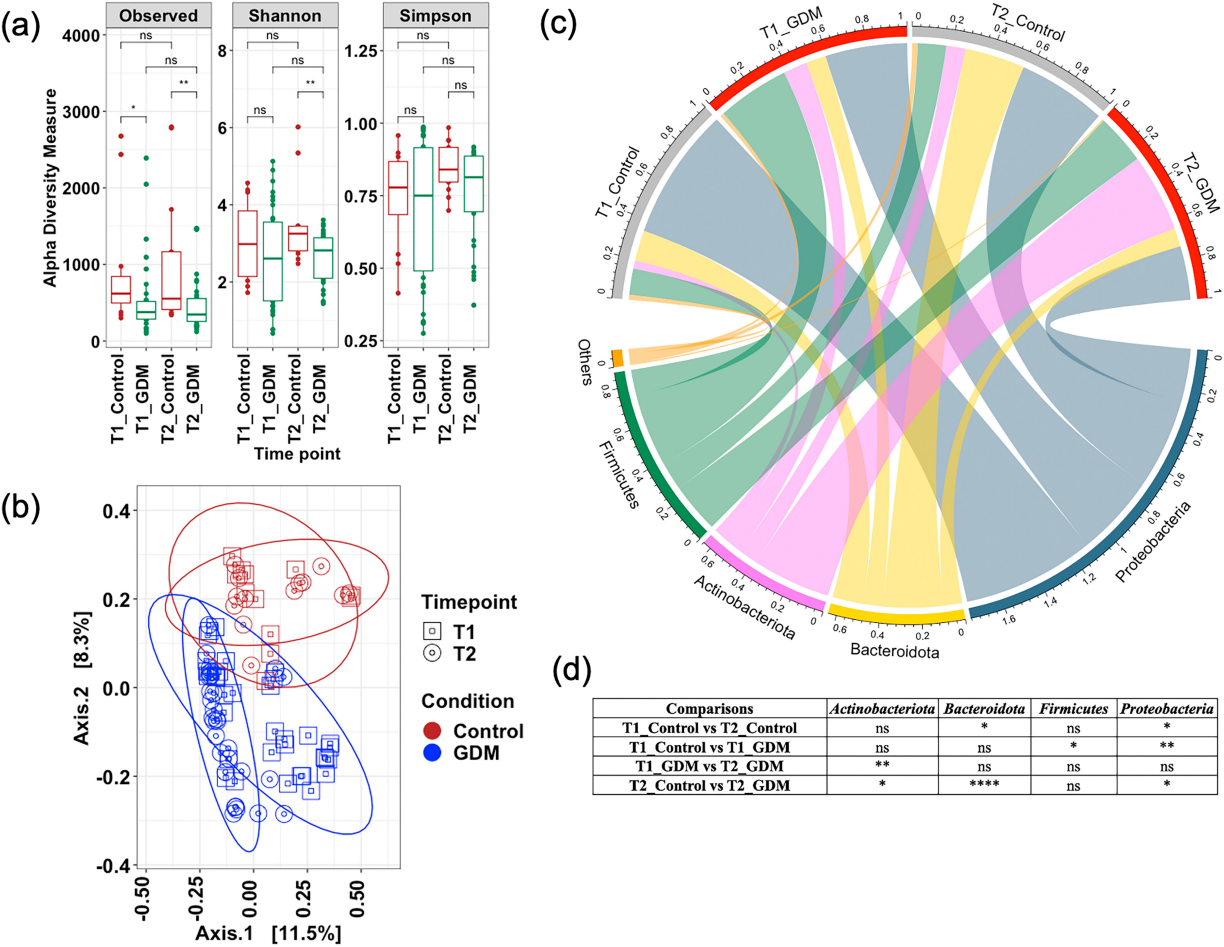
Microbiota composition of meconium and stools from six‐week‐old infants. (a) Alpha diversity measures of meconium (T1) and stool (T2) samples of infants born to GDM and control mothers. Statistical significance is assessed by Pairwise Wilcoxon test. (b) Principal coordinate analysis plot represents the microbiome profile of T1 and T2 stool samples of infants born to GDM and control mothers. (c) Circlize plot represents the distribution of major bacterial phylum in T1 and T2 stool samples of infants born to GDM and control mothers. (d) Pairwise statistical comparisons of bacterial phylum using Wilcoxon test on meconium (T1) and stools (T2) samples of infants born to GDM and control mothers.* denotes *p* ≤ 0.05, ** denotes *p* ≤ 0.01, *** denotes *p* ≤ 0.001, **** denotes *p* ≤ 0.0001, ns (not significant) denotes *p* > 0.05.

Alpha diversity measures revealed that infants born to GDM mothers had a lower number of microbial taxa (Wilcoxon test, *p <* 0.05) than control infants at both time points (Figure 2a). The species richness was found to be significantly (*p <* 0.05) reduced from T1 to T2 in infants born to GDM mothers as compared to their control counterparts (Figure 2a). The differences in microbiome profile between infants born to GDM mothers and control mothers were reflected through PCoA‐based analysis (Figure 2b). Pairwise PERMANOVA analysis showed that T1 stool samples of infants born to GDM mothers were significantly (*p <* 0.05) different from T1 stool samples of infants born to control mothers. Similarly, significant (PERMANOVA, *p <* 0.05) differences in microbial composition were also noted between T2 stool from infants born to GDM mothers and those born to control mothers (Figure 2b).

Phylum level composition revealed a stark difference in the microbiome profile in T1 and T2 stool samples in these two cohorts (infants born to GDM mothers and infants born to control mothers) (Figure 2c). In control infants (i.e., born to non‐GDM mothers), *Proteobacteria* was the dominant member of the T1 population, followed by *Bacteroidota*, *Firmicutes*, and *Actinobacteriota* (Figure 2c). In T1 stool samples of infants born to GDM mothers, the dominant microbiome was *Firmicutes* and *Proteobacteria*. Their abundance in *Firmicutes* members was more than that of their control counterparts (*p <* 0.05) (Figure 2c,d), while their abundance of *Proteobacteria was* less (*p <* 0.05). The T2 stool microbiome profile of infants born to GDM mothers had a higher abundance of *Actinobacteriota* (*p <* 0.05) and a lower abundance of *Bacteroidota* (*p <* 0.05) and *Proteobacteria* (*p <* 0.05), compared to T2 stool samples of control infants (Figure 2c,d). When T2 stool samples of the control infants were compared against their T1 stool samples, there was a significant increase in the abundance of *Bacteroidota* (*p <* 0.05) and a significant reduction in the abundance of *Proteobacteria* (*p* < 0.05) (Figure 2c,d). When the T2 stool samples of infants born to GDM mothers were compared against their T1 stool samples, there was a significant increase in *Actinobacteriota* (*p* < 0.05) and a significant decrement in *Firmicutes* and *Proteobacteria* populations (Figure 2c,d).

We further tried to understand the changes in the microbiome pattern in relation to the feeding habits of the infants. Our results suggested that at one month of life (T2), infants who were on exclusive breast feeding did not show a significant difference (pairwise PERMANOVA analysis: *p*.adjusted = 0.159 for infant born to GDM mothers and *p*.adjusted = 0.55 for infant born to control mothers) in their microbial community composition with those on mixed feeding (i.e., fed both breast milk and formula feeds), irrespective of their GDM status (Figure S1).

We also tried to study the impact of perinatal antibiotic usage in mothers with GDM on the gut microbiome of their infants at T2 timepoint. The results did not yield a significant difference in the infant gut microbiome profile between mothers exposed to antibiotics and control mothers (no antibiotic exposure) (Figure S2).

### 3.3 Differentially Abundant Bacterial Signatures in T1 and T2 Stool Samples of Infants

We further analyzed the differentially abundant microbial taxa (ASVs) in T1 and T2 stool samples of infants born to GDM and control mothers (Figure S3). For T1 samples, DeSeq2 based analysis identified 78 microbial ASVs (*p* < 0.001) which were differentially abundant in infants born to GDM mothers (Figure S3a). These enriched ASVs were mostly affiliated to *Bifidobacterium*, *Blautia*, *Collinsella*, *Clostridium sensu stricto* 1, *Acinetobacter*, *Bacteroides*, *Pseudomonas*, *Staphylococcus*, *Enterococcus*, *Eubacterium halli group*, *Erysipelotrichaceae* UCG‐003, and *Sellimonas*. In contrast, ~600 ASVs were found to be differentially abundant in T1 stool samples of control infants (Figure S3a). These ASVs were associated with *Escherichia*‐*Shigella*, *Bacteroides*, *Sutterella*, *Prevotella* 9, *Sphingomonas*, *Solirubrobacter*, *Gaiella*, *Veillonella*, *Parabacteroides*, *Alistipes*, *Lachnospiraceae NK4A136 group*, and *Haemophilus*.

For T2 samples, ~123 ASVs were found to be differentially abundant in T2 stool samples of infants born to GDM mothers, while ~660 ASVs were differentially abundant in control infants (Figure S3b). *Bifidobacterium*, *Bacteroides*, *Blautia*, *Intestinibacter*, *Rothia*, *Clostridium sensu stricto* 1, *Staphylococcus*, *Eubacterium hallii* *group*, *Streptococcus*, *Ruminococcus torques* *group*, etc. were enriched in T2 stool samples of infants born to GDM mothers. On the contrary, *Escherichia*‐*Shigella*, *Bacteroides*, *Prevotella* 9, *Parabacteroides*, *Bacillus*, *Alistipes*, *Sutterella*, *Sphingomonas*, *Gaiella*, *Conexibacter*, *Streptomyces*, *Bryobacter*, etc. were enriched in T2 stool samples of control infants.

### 3.4 Predictive Metabolic Profiling of T1 and T2 Stool Samples of Infants

Predicted functional pathways of the T1 and T2 stool microbiome of infants born to GDM and control mothers were determined through PICRUSt2. Differentially enriched pathways were identified in both T1 and T2 stool samples (Figure S4). The results revealed that T1 stool samples from control infants were enriched with lipopolysaccharide biosynthesis, cyanoamino acid metabolism, glutathione metabolism, and two‐component system as compared to its counterpart (Figure S4a). On the contrary, T2 stool microbiome of infants born to GDM mothers showed enrichment in pentose phosphate pathway, cysteine and methionine metabolism, glycolysis/gluconeogenesis, and glycine, serine, and threonine metabolism as compared to control infants (Figure S4b). In addition, starch and sucrose metabolism, selenocompound metabolism, and C5‐branched dibasic acid metabolism were also enriched in T2 stool samples from infants born to GDM mothers compared to its counterpart.

### 3.5 Comparison of Mothers' Microbiome With T1 and T2 Infant Stool Samples

**FIGURE 3:**
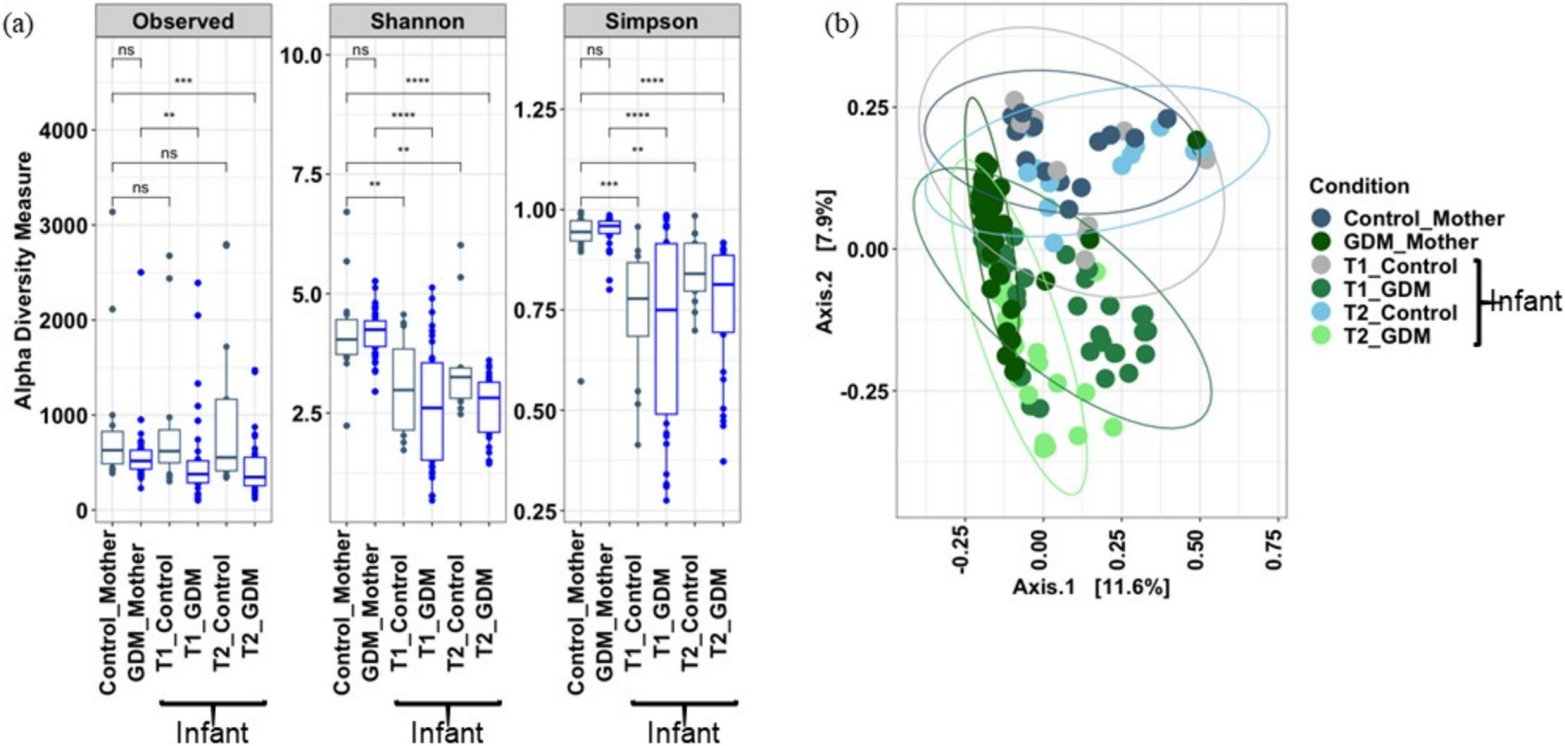
Comparison of gut microbiome of mothers with meconium and stool samples from infants. (a) Alpha diversity measures of control and GDM mothers with their respective offspring's T1 and T2 stool samples. Statistical significance is assessed by Pairwise Wilcoxon test. (b) Principal coordinate analysis plot represents the microbiome profile of control and GDM mothers with their respective offspring's meconium (T1) and stools (T2) samples. * denotes *p* ≤ 0.05, ** denotes *p* ≤ 0.01, *** denotes *p* ≤ 0.001, **** denotes *p* ≤ 0.0001, ns (not significant) denotes *p* > 0.05.

**FIGURE 4:**
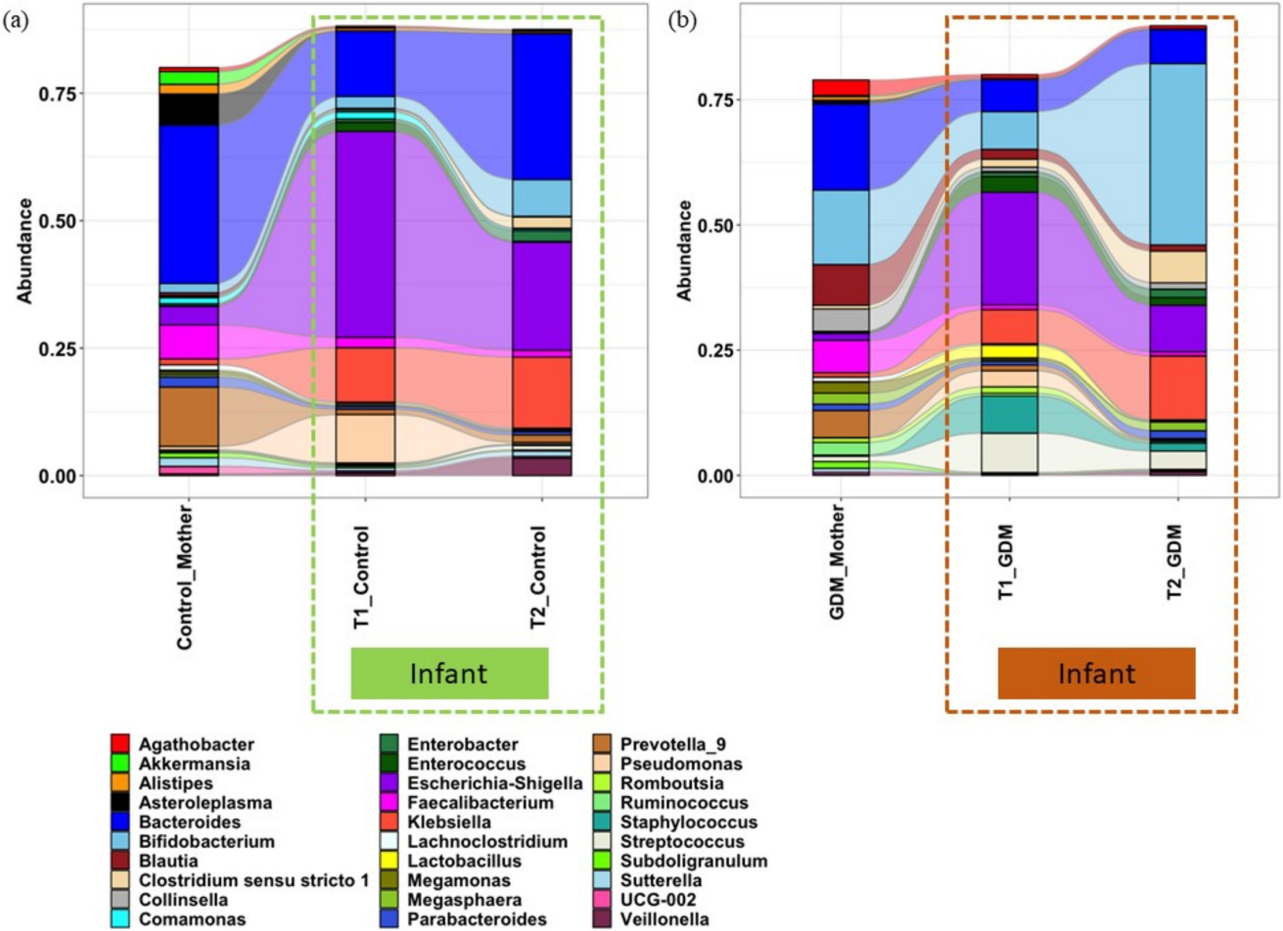
Alluvial flow diagram shows the distribution of major bacterial genera in the gut microbiome of mothers and their respective offspring's T1 and T2 stool samples. (a) Distribution of major genera in GDM cohort. (b) Distribution of major genera in control cohort.

Alpha diversity measures showed that the mothers had significantly (*p <* 0.05) higher microbial richness and evenness (in terms of Shannon diversity index) compared to their infants (Figure 3a). No significant difference in microbiome profile between the control mothers and their infants [PERMANOVA analysis, T1 stools versus control mothers (*p* = 0.53), T2 stools versus control mothers (*p* = 0.29)] was observed (Figure 3b). When the microbiome profile of the GDM dyads was compared to the control dyads, a clear‐cut distinction was observed (Figure 4a,b). Mothers with GDM had a higher abundance of *Bifidobacterium*, *Collinsella*, *Blautia*, *Ruminococcus*, *Agathobacter*, *Streptococcus*, *Megasphaera*, *Clostridium sensu stricto* 1, and *Enterococcus*, compared to control mothers. These microbial taxa contributed major proportions in T1 and T2 stool samples in infants born to mothers with GDM. The alluvial flow diagrams also showed the changes and development of the infant's gut microbiome from T1 to T2 (Figure 4a,b). Based on the BMI level, we divided mothers into four categories (i) level‐1 (below 18.5), (ii) level‐2 (18.5–24.9), (iii) level‐3 (25.0–29.9), and (iv) level‐4 (above 30). We found that most of the mothers belonged to level‐2 and level‐3 categories. However, we could not find the significant difference in microbial composition based on their BMI level (Figure S5).

### 3.6 Vertical Transfer of Core Microbiome From Mothers to Infants

To further understand the vertical transmission event of gut microbial taxa from mothers to their offspring in seeding of early life microbiota, we identified the core gut microbiome members of the mothers and their trans‐generational transfer (Figure S6). For control mothers, 30 ASVs were found to be a part of the core microbiome, while 44 ASVs were identified as core microbiome members in mothers with GDM. The transfer of these microbial members in T1 and T2 of life in the respective cohorts was clear through heatmap‐based analysis (Figure S6a,b). ASVs, such as *Faecalibacetrium*, *Escherichia*‐*Shigella*, *Bacteroides*, *Acidaminococcus*, and *Parabacteroides*, which contributed substantially to the gut microbiome of control mothers, were transferred and retained in varying abundance till 6 weeks of life in their respective offspring. On the contrary, ASVs belonging to *Bifidobacterium*, *Blautia*, *Collinsella*, *Bacteroides*, *Romboutsia*, and *Agathobacter* were transferred from GDM mothers to their infants.

### 3.7 Transfer of GDM Associated Bacterial Signatures From Mothers to Infants

Transgenerational transfer of GDM‐associated bacterial biomarker from mother to infant during early life microbiota was investigated by identifying the differentially enriched or abundant microbial taxa in GDM mothers. DeSeq2‐based analysis identified nearly 253 ASVs which were differentially abundant in GDM mothers (Table S1). Out of these 253 ASVs, we considered only 127 ASVs for this analysis (those whose log2 fold change was higher than 3.5 with *p*.adjusted < 0.001 for more robust analysis). Vertical transmission event confirmed the transfer of GDM‐associated ASVs (such as *Blautia*, *Collinsella*, *Bifidobacterium*, *Romboutsia*, *Clostridum sensu stricto* 1, *Anaerostipes*, *Intestinibacter*, *Ruminococcus*, *Streptococcus, Dorea*, and *Eubacterium halli group*) in infants born to mothers with GDM and persisted at 6 weeks of life (Figure S7). NMDS analysis further supported the difference in the abundance profile of these GDM associated ASVs in T1 and T2 stool samples from infants born to mothers with or without GDM (Figure S8).

### 3.8 Source Proportion and Transfer of ASVs in Mother‐Infant Dyad Pairing

**FIGURE 5:**
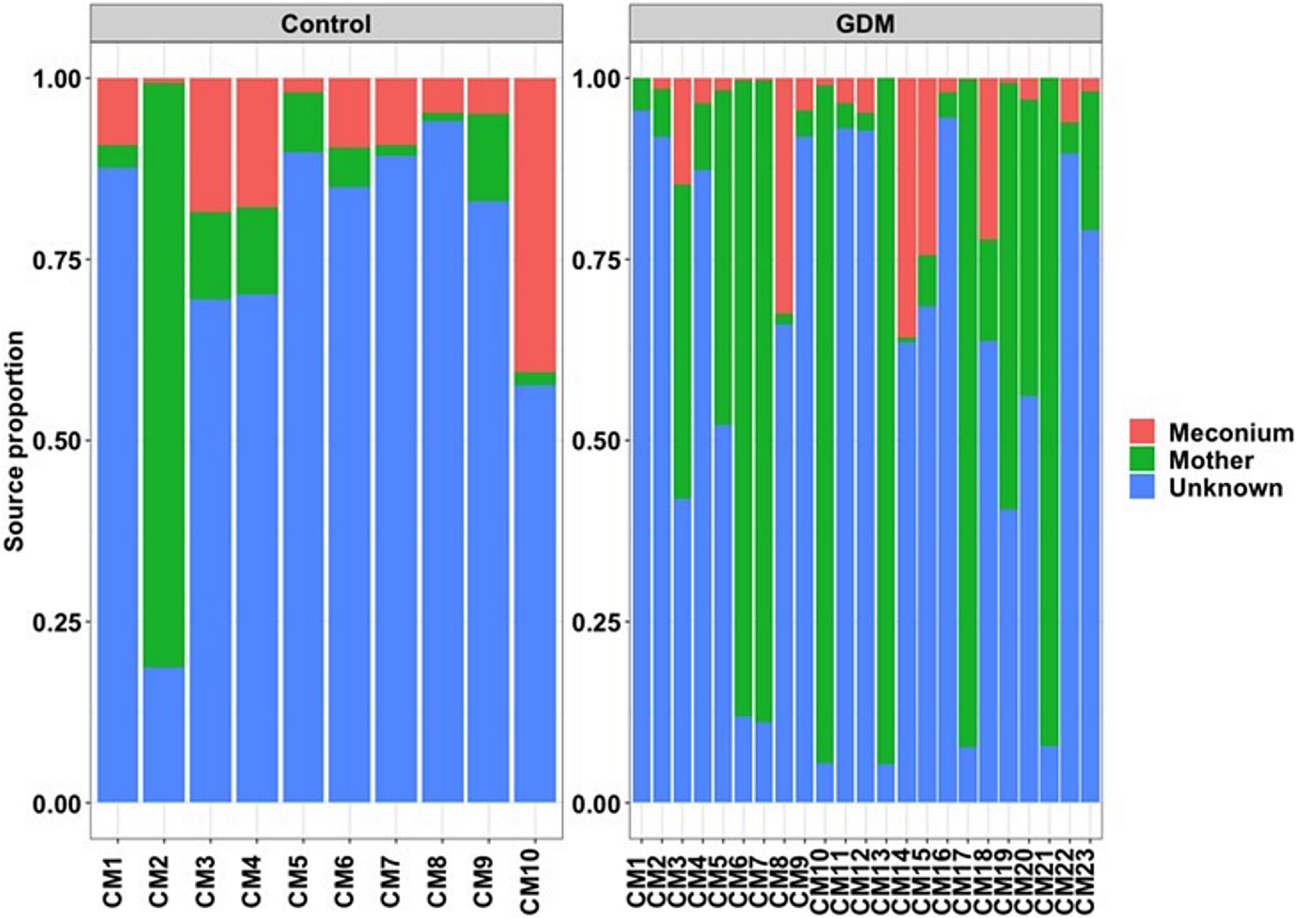
The proportion of sources in the infant's stool (6 weeks old) from their respective mother‐infant dyad pairings using FEAST.

To develop a more detailed understanding of the vertical transmission events, we limited the analysis to mother‐infant dyad pairs delivered through the vaginal route, for whom both T1 and T2 stool microbiome datasets were available. In total, 23 mother‐infant dyad pairs were used for this analysis from the GDM cohort, whereas 10 mother‐infant pairs were considered from the control cohort. Our source tracking analysis using FEAST identified that the relative contribution of mothers was much higher than that of meconium (T1) in stool samples of infants (T2) born to GDM mothers. The source proportion was also found to vary among the mother‐infant pairs (Figure 5).

**FIGURE 6:**
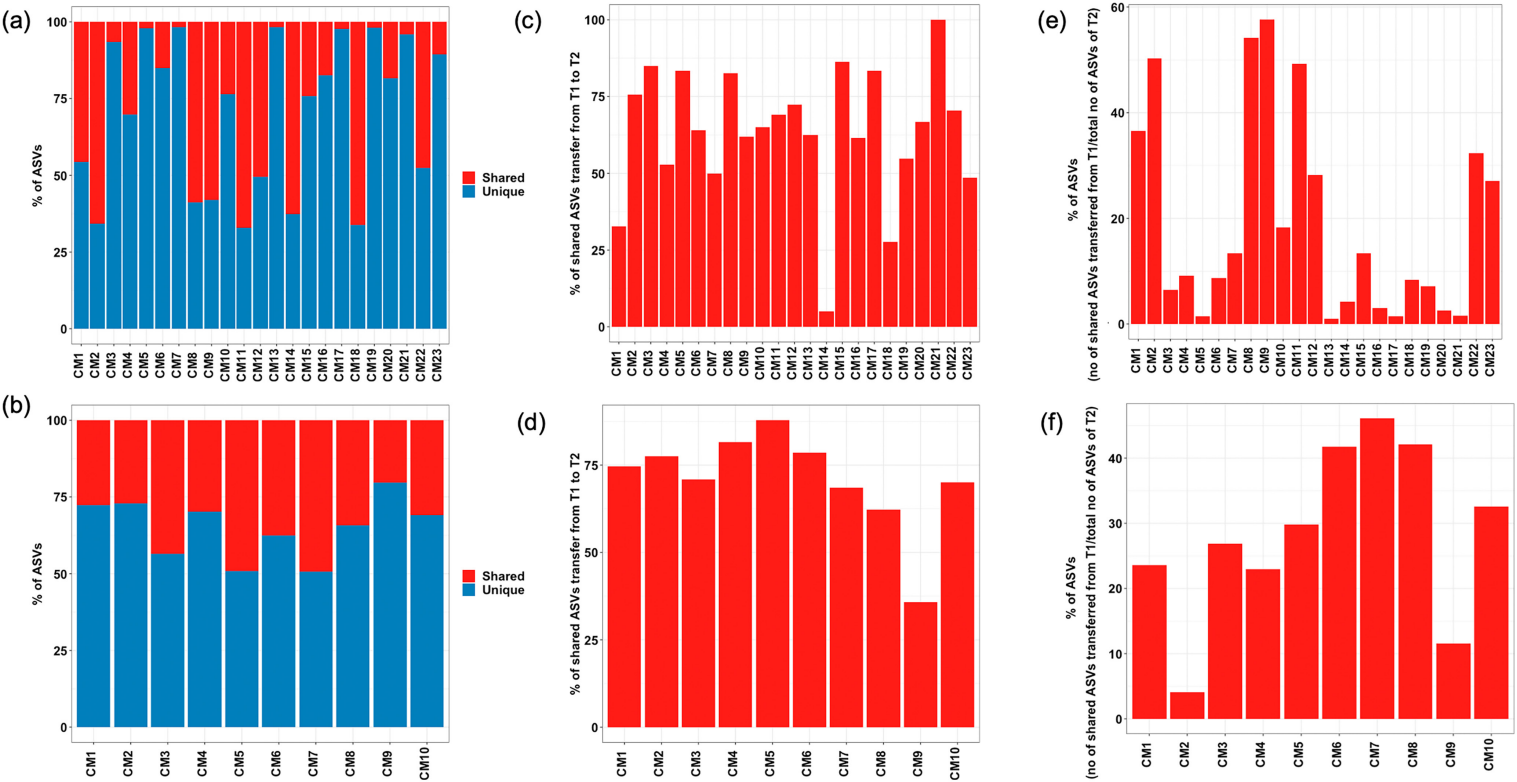
Transfer and retention of total microbes in mother‐infant dyad pairing. % of shared (with mothers) and unique (not shared) ASVs in meconium samples of infants born to (a) GDM mothers and (b) control mothers, in their respective dyad pairings. Transfer of shared ASVs present in meconium samples to T2 stool samples of infants born to (c) GDM mothers and (d) control mothers, in their respective dyad pairings. Contribution of shared ASVs transferred from meconium to T2 stool samples in infants born to (e) GDM mothers and (f) control mothers, in their respective dyad pairings.

We further identified the shared ASVs in each mother‐infant pair between the gut microbiome of mothers and T1 stool samples. The results revealed that T1 stool samples of infants born to GDM mothers in each mother‐infant pair shared 1.63%–67.22% of ASVs from their mother's gut microbiome (Figure 6a). On the other hand, infants born to control mothers in their respective mother‐infant pair dyads shared 20.25%–49.28% of ASVs from their mother's gut microbiome (Figure 6b).

Next, we evaluated the proportion of these shared ASVs from mothers in the infants' T1 stool samples which were transferred to the infants' T2 stool samples. For the GDM cohort, the results showed that out of the total shared ASVs in meconium from their mothers, more than 48% (up to 100%) of them were transferred to each of the T2 stool samples, except three samples (ranged from 4.96% to 32.72%) (Figure 6c). For the control cohort, more than 60% (up to 87.84%) of the total shared ASVs in meconium were transferred to each of the T2 stool samples except for one sample (35.86%) (Figure 6d).

We also investigated the percentage contribution of these shared ASVs (transferred from meconium to T2 stools) in T2 stools. The results demonstrated that these transferred ASVs from meconium contributed up to 57.6% of total ASVs of T2 stool samples in the GDM cohort, while in the control cohort, they constituted up to 46.1% of total ASVs (Figure 6e,f).

Last but not least, we examined if there were any GDM‐associated ASVs which were frequently shared by mother‐infant dyad pairs in the GDM cohort (Figure S9). We found that ASVs affiliated to *Fusicatenibacter* (ASV31) and Streptococcus (ASV20) were frequently detected in mother‐infant pairs (*n* = 19) followed by *Collinsella* (ASV6, *n* = 18), *Blautia* (ASV7, *n* = 18), Dorea (ASV76, *n* = 17), *Agathobacter* (ASV15, *n* = 16), *Romboutsia* (ASV35, *n* = 16), *Bifidobacterium* (ASV8, *n* = 16), *Blautia* (ASV42, *n* = 15), and *Ruminococcus gnavus* *group* (ASV75, *n* = 15). Additionally, out of the total shared ASVs in T1 samples from their respective mother across the dyad pairings, GDM‐associated bacterial ASVs in these shared ASVs (identified through DeSeq2 based analysis) constituted up to 21.5% and 18.25% of the total ASVs in T1 and T2 samples, respectively.

## 4 Discussion

In this prospective cohort study, we showed that infants born to GDM mothers had distinctive early life microbiomes (meconium and six weeks old stools) compared to infants born to non‐GDM mothers (PERMANOVA, *p*.adjusted < 0.05) using targeted 16S rRNA gene amplicon sequencing of stool samples of pregnant GDM and non‐GDM women coupled with paired meconium and 6‐week stool samples of their infants through source tracking.

We also demonstrated that the microbial community composition of meconium of infants born to non‐GDM (control) mothers was less diverged and had distinct microbial profiles from infants born to GDM mothers. This observation is in concordance with previous reports where meconium samples of infants born to non‐GDM mothers or healthy mothers mainly constituted *Proteobacteria* with predominance of *Escherichia‐Shigella, Enterococcus, Pseudomonas, and Klebsiella* [24, 25, 26, 27]. However, in the present study, meconium samples of infants born to GDM mothers reflected mothers' microbiome, with the presence of key microbes such as *Blautia, Collinsella, Eubacterium halli group, Ruminicoccus, and Bifidobacterium*, which are known to be involved in insulin resistance and proinflammatory conditions during pregnancies with GDM [6, 7, 8, 9, 10]. Interestingly, the microbiome profile of the stool samples after six weeks of life changed substantially in the infants, alluding to the role of potentially modifiable intrinsic and extrinsic factors such as host metabolism, external environment, type of milk feeding (i.e., breastfeeding/formula‐fed milk) [12, 28, 29]. In our dataset, it is regrettable that we could not demonstrate any significant differences in gut microbiome of infants who received exclusive breastfeeding versus mixed feeds. This is likely related to the small sample size of patients recruited for this pilot study. This is certainly a limitation of our study, and we acknowledge that larger numbers would be helpful to understand if exclusive breastfeeding in this vulnerable population could be a modifiable factor to reduce gut dysbiosis in infants born to GDM mothers.

Compared to infants of control mothers, we also found that the metabolic functions of the bacterial species in infants born to GDM mothers showed differences in the enrichment of the pentose phosphate pathway, cysteine and methionine metabolism, glycolysis/gluconeogenesis, and glycine, serine, and threonine metabolism. These results indicate that alteration in the aforementioned metabolism in infants is possibly linked to maternal metabolic status (as pregnant women with GDM have shown enrichment of these pathways) [6, 7, 8, 9, 10]. These results further support the notion that infants born to GDM mothers have different metabolic status compared to infants born to mothers with no GDM. Larger studies looking into these pathways are important to better understand how transgenerational gut dysbiosis could contribute to the development of childhood metabolic disorders in the near future.

In addition, this longitudinal study helps to provide evidence of mother to infant vertical transgenerational microbiome transference patterns in GDM. More significance should be placed on understanding the formation of the early‐life microbiome, as this could affect an infant's physiology and future health in future work. Till date, there is limited evidence on the prediction of mother‐infant gut microbiota in the long‐term outcomes of mother‐infant dyads with GDM [9]. We believe that this can be addressed with larger cohort studies, which may then guide us to design specific interventions, including the use of probiotic supplementation for mother and infant dyads with dysbiosis related to GDM in the future.

Interestingly, we also observed that there was a greater transfer and retention of total microbes and associated GDM bacterial signatures in mother‐infant dyads with GDM compared to healthy mother‐infant dyads. This was evidenced by vertical transmission events which confirmed the transfer of GDM associated ASVs (such as *Blautia*, *Collinsella*, *Bifidobacterium*, *Romboutsia*, *Clostridium sensu stricto* 1, *Anaerostipes*, *Intestinibacter*, *Ruminococcus*, *Streptococcus*, *Dorea*, *Eubacterium halli group*) in infants born to mothers with GDM and retained at 6 weeks of life. The significance of this is less known in early life microbiome and merits further work in large prospective cohort studies to understand the importance of these vertically transferred microbes from mother to infant and their impact on host metabolism and immunity. Soderborg and colleagues [11, 30] have reported that GDM with maternal obesity led to the alteration in the colonization of the infant gut microbiome and reflected much similar profiles to their respective mothers, indicating that maternal microbiota is the source of first colonizers in the infant gut.

Similarly, Crusell et al. [31] observed that glycaemic regulation in late pregnancy was also found to be linked with the variation in the gut microbiota structure of the offspring till 9 months of infants' life. Furthermore, they identified that these alterations in the microbiome of offsprings born to GDM mothers showed similarities with the gut microbiome detected in childhood obesity and type 2 diabetes in adults. Several other studies also identified the vertical transmission event of microbes from mother to infants in various diseases and disorders and its importance in the alteration of infant metabolism, physiology, immunity, and growth and development [30, 32, 33, 34, 35]. Moreover, maternal diet during pregnancy affects neonatal early life microbial colonization and contributes substantially to infant development, indicating the need for specific dietary programs during pregnancy that promote adequate transmission of healthy microbes from mother to their infants [36].

Despite the modest size of our cohort, our study has strengths. The strengths of our cohort include the strict inclusion criteria (as detailed in the Methods section under recruitment) to reduce various, often‐overlooked known clinical covariates (such as maternal BMI, antibiotic interventions for mother and infants, and mode of delivery) and the presence of a healthy mother‐infant cohort for comparison as well as follow‐up to 6 weeks of life post‐partum.

The limitations of our study include the inability to exclude the effect of other potential confounders such as clinical (i.e., lifestyle and diet). We acknowledge that the study could have benefited from incorporating comprehensive dietary and lifestyle assessment techniques so as to differentiate shifts in microbiome between GDM‐associated ones and lifestyle or diet‐associated ones. Future work should collect comprehensive details of dietary requirements to allow for a more holistic analysis. In addition, this study did not have a diverse spread of ethnicity. However, we have previously demonstrated that ethnicity does not significantly affect the maternal microbiota [13]. We also did not collect maternal stools in the first trimester of pregnancy, which could limit generalizability as immune and metabolic changes can occur throughout pregnancy, and the gut microbiota shifts from the first to third trimester [34]. We did not have the metabolic data of the infants and mothers to uncover how the gut microbiome modulates the metabolic pathways in these conditions. While some studies have shown that there could be microbiome changes across the trimesters, others have demonstrated that gut microbiota remain stable, including our earlier paper [13] which showed that maternal microbiota did not change during the 2 periods. A larger prospective cohort of pregnant women will help to address these questions. Future work should include metatranscriptomics and shotgun‐metagenomics which would have provided microbial species‐level information and detailed changes in the metabolic pathways in the early life microbiome. Finally, this is a small pilot study with a relatively modest sample size and a short follow‐up period. Further studies should include longer follow‐up to track microbiome maturation and health outcomes up to 2 years of age.

In conclusion, our results demonstrate that early life microbiome is dependent upon the health status of the mother as evidenced through vertical transmission of GDM‐associated markers in infants born to GDM mothers. It also highlights the importance of understanding the transgenerational effect from mother to child and the formation of early‐life microbiome, which could affect an infant's physiology and future health.

## Author Contributions

L.Y.L., J.M.L., S.Y.C., S.L.L., and S.S. conceived and designed the study. J.M.L., L.Y.L., and R.T. collected the clinical data. L.Y.L. and S.S. supervised the study. A.G. performed the bioinformatic analysis with inputs from L.Y.L., J.M.L., S.S., and S.Y.C. J.M.L., A.G., and L.Y.L. wrote and edited the manuscript with substantial support from S.Y.C., S.L.L., S.S., and R.T. J.M.L., R.T., and L.Y.L. recruited the subjects and collected the specimens. All authors critically reviewed and approved the final manuscript.

## Disclosure

The authors have nothing to report.

## Ethics Statement

This study was approved by the National Healthcare Group Domain Specific Review Board (NHG DSRB), Singapore (Reference no.: 2019/00064).

## Consent

Informed consent was obtained from all individual participants included in the study.

## Conflicts of Interest

The authors declare no conflicts of interest.

## Supporting information


**Data S1:** Supporting Information.

**Table S1:** GDM associated markers used for identification of its transfer in infants.

## Data Availability

The data that support the findings of this study are available in NCBI under Bioproject number PRJNA945212, and within the article and its electronic supporting information.
