# Supplementary material for: Vertical Transmission of Gut Dysbiosis From Mothers With Gestational Diabetes to Infants
Source: J Diabetes. 2025 Oct 1;17(10):e70148. doi: 10.1111/1753-0407.70148 (PMC12488368; doi:10.1111/1753-0407.70148)
Supplement: Supplementary file 1 — Data S1: Supporting Information. [file JDB-17-e70148-s002.docx]

**Supplementary Material**

**Vertical transmission of gut dysbiosis from mothers with gestational diabetes to infants**

*Running Title:* Gut dysbiosis transmission in GDM

Jia Ming Low^1,2†*,^ Abhishek Gupta^3†*^, Rachel Toh^4^, Su Lin Lim^5^, Shiao-Yng Chan^6^, Sanjay Swarup^3,7,8^, Le Ye Lee^1,2^

^1^ Department of Neonatology, Khoo Teck Puat-National University Children Medical Institute, National University Hospital, National University Health System, Singapore

^2^ Department of Paediatrics, Yong Loo Lin School of Medicine, National University of Singapore, Singapore

^3^ Singapore Centre for Environmental Life Sciences Engineering (SCELSE), National University of Singapore, Singapore

^4^ Department of Paediatrics, Khoo Teck Puat-National University Children Medical Institute, National University Hospital, National University Health System, Singapore

^5^ Office of Allied Health & Pharmacy, National University Hospital, National University Health System, Singapore

^6^ Department of Obstetrics and Gynaecology, National University Hospital, National University Health System, Singapore

^7^ Department of Biological Sciences, National University of Singapore, Singapore

^8^ NUS Environmental Research Institute (NERI), Singapore

*^†^These authors contributed equally.*

**Corresponding Authors:*

1. **Jia Ming Low, MBBS, MMed (Paeds), MRCPCH, FAMS, MCI**

Department of Neonatology, Khoo Teck Puat – National University Children's Medical Institute, National University Hospital, National University Health System, Singapore, Singapore

Department of Paediatrics, Yong Loo Lin School of Medicine, National University of Singapore, Singapore

1E Kent Ridge Road, NUHS Tower Block Level 12, Singapore 119228

Email: [paeljm@nus.edu.sg](mailto:paeljm@nus.edu.sg)

Phone: (65) 6772 5075

1. **Abhishek Gupta, PhD**

Singapore Centre for Environmental Life Sciences Engineering, National University of Singapore, Singapore

60 Nanyang Dr, Singapore 637551

Email: [a_gupta7@nus.edu.sg](mailto:a_gupta7@nus.edu.sg)


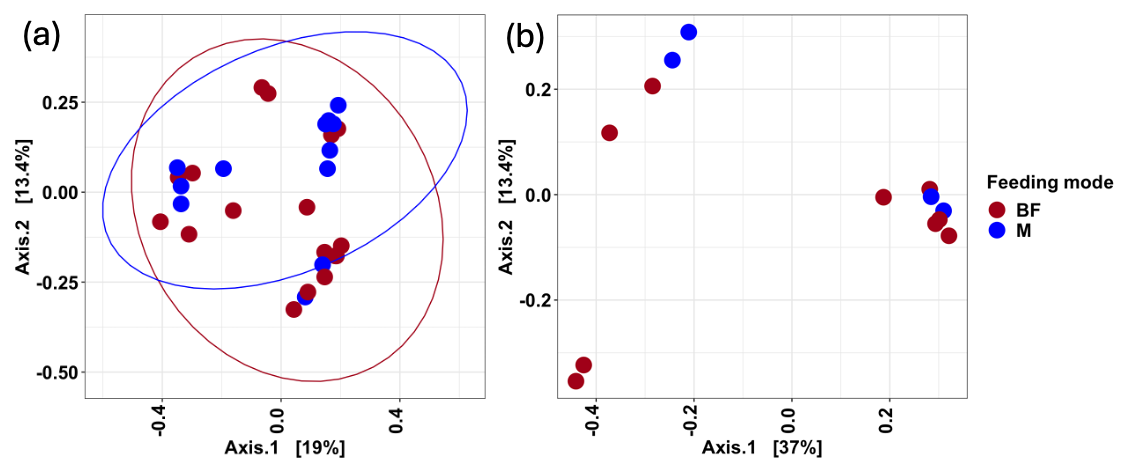


**Supplementary Figure S1.** Principal coordinate analysis plot represents the microbiome profile of infants (at T2 timepoint) with different feeding habits. (a) Infant born to GDM Mothers and (b) Infant born to control mothers. BF, F, M denotes exclusively breastmilk feeding, formula feed, and mixed feeding, respectively.


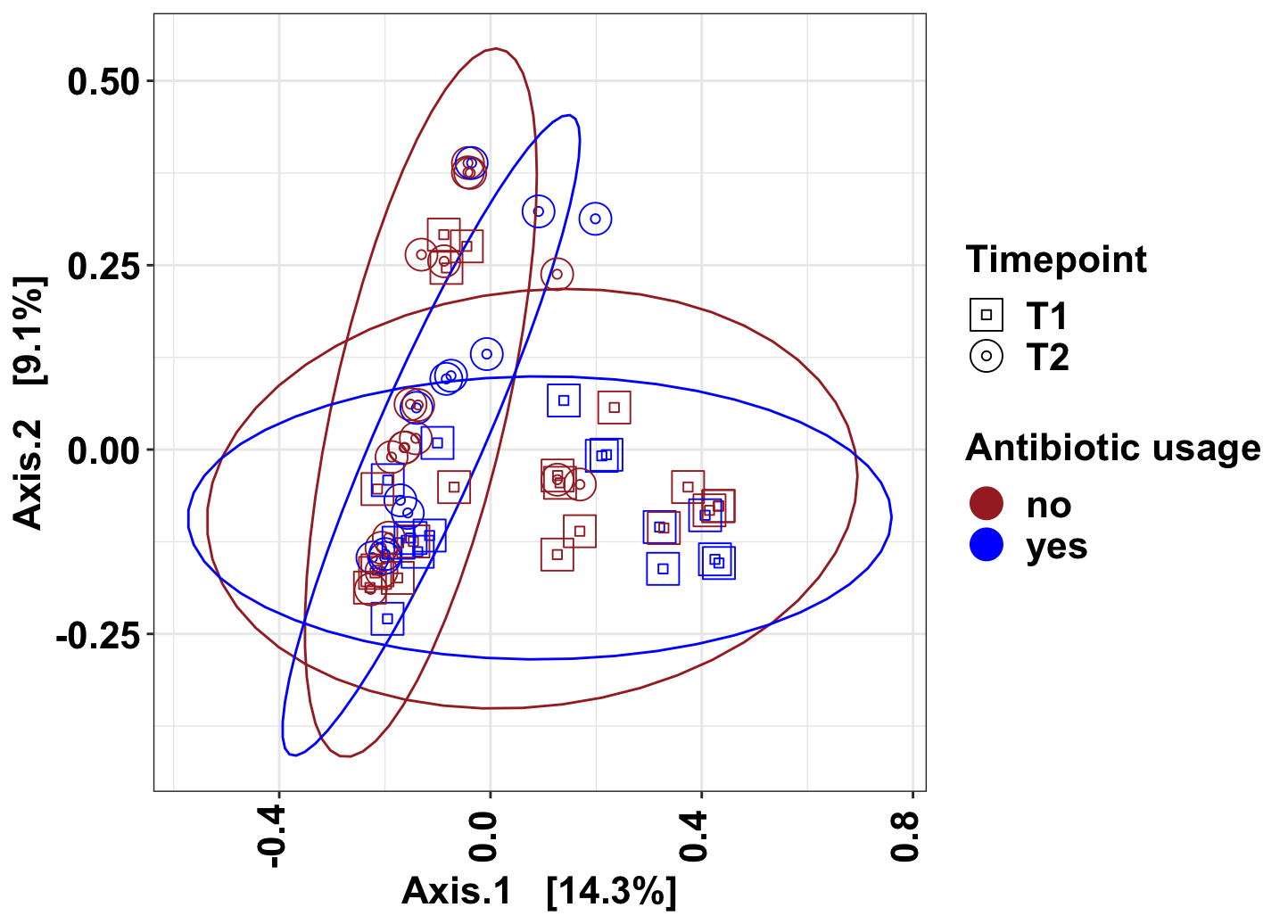


**Supplementary Figure S2.** Principal coordinate analysis plot represents the impact of perinatal antibiotic exposure to GDM mothers on gut microbiome profile of their infants Pairwise PERMANOVA analysis for also performed to assess the change in their microbial community analysis. All the combinations are represented for pairwise PERMANOVA analysis (noT1 vs noT2: p.adjusted =0.0195, noT1 vs yesT1:p.adjusted =0.2150, noT1 vs yesT2: p.adjusted =0.0120, noT2 vs yesT1:p.adjusted =0.0060, noT2 vs yesT2:p.adjusted =0.2052, yesT1 vs yesT2:p.adjusted =0.0120)


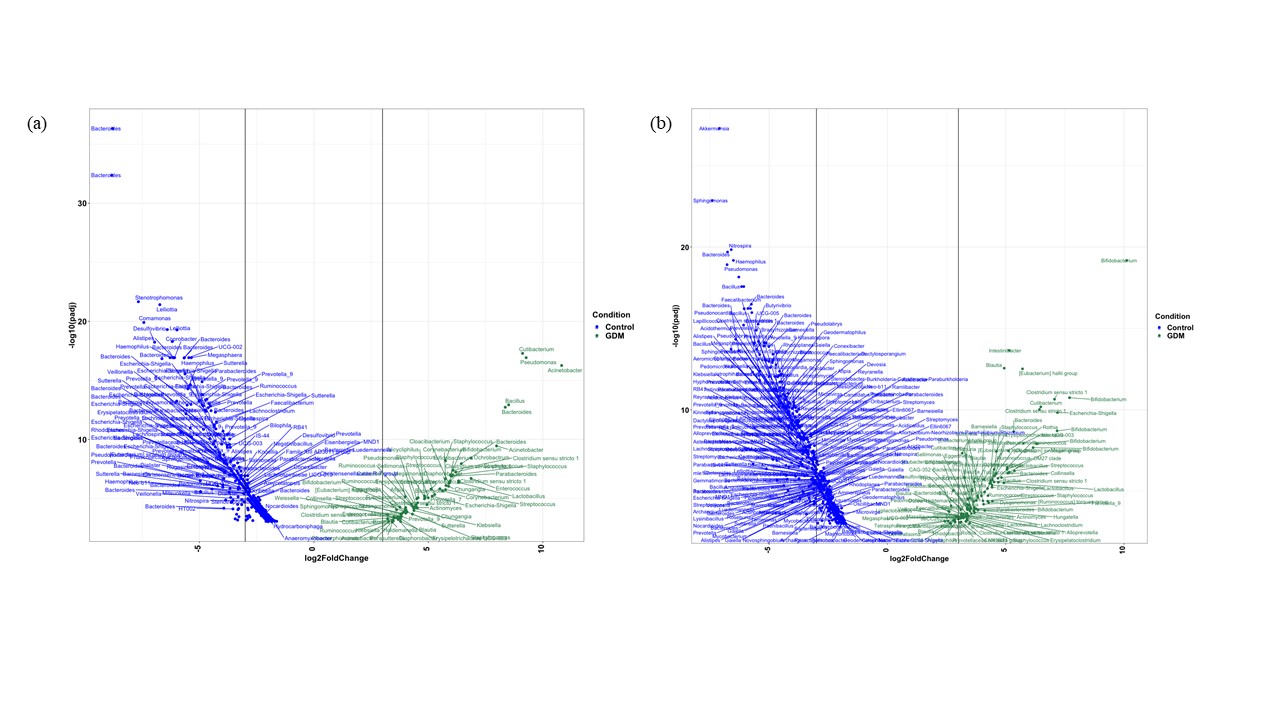


**Supplementary Figure S3.** Volcano plots represent the differential abundant microbial taxa (ASVs) identified through DeSeq2 analysis. (a) Differentially abundant ASVs in meconium samples of neonates born to GDM and control mothers. (b) Differentially abundant ASVs in stool samples of infants born to GDM and control mothers.


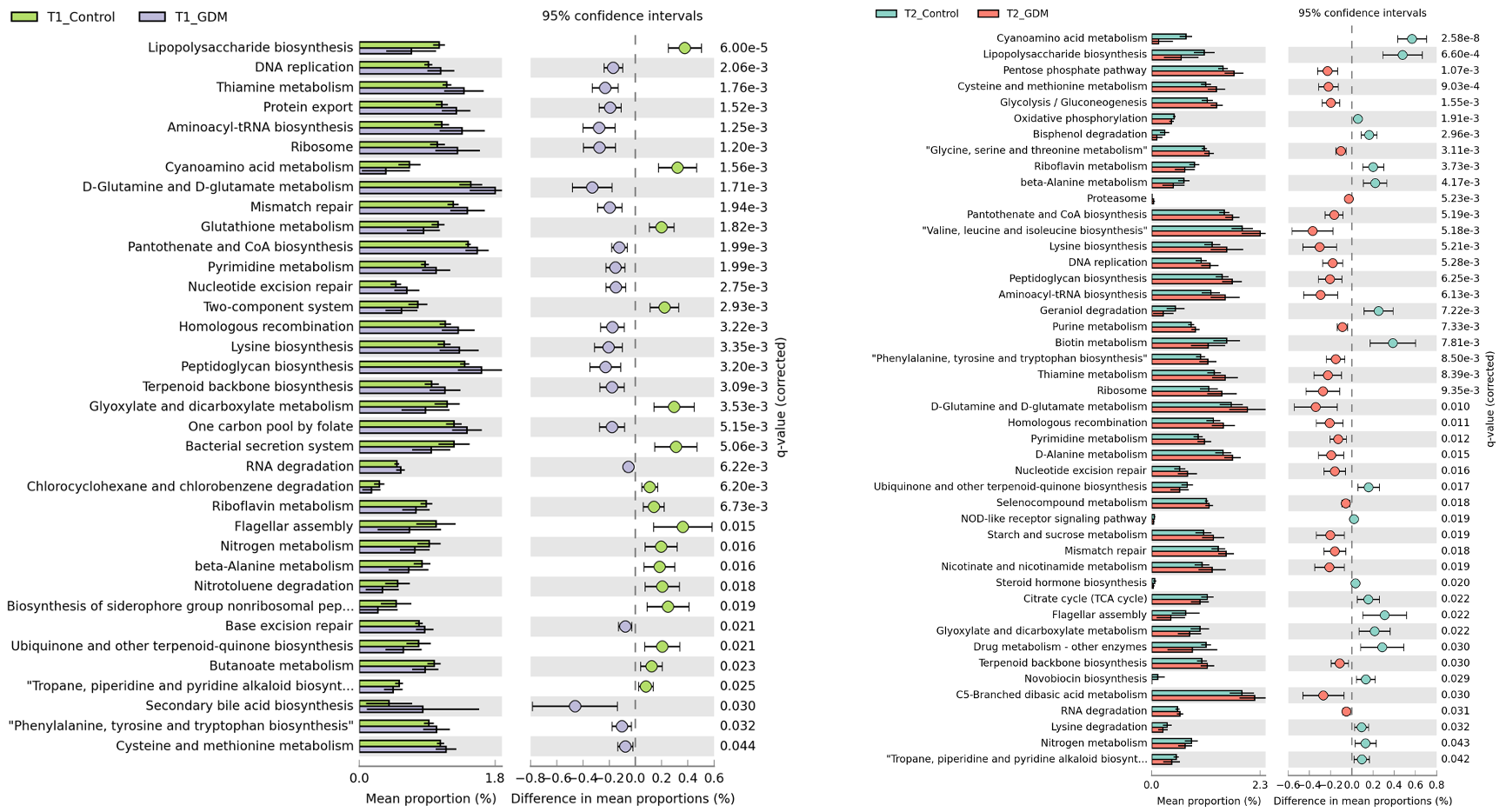


**Supplementary Figure S4.** Extended error bar plot (with *P* < .05) for two-group analysis module comparison of PICRUSt predicted KEGG function data based on meconium and stool samples from infants born to (a) control mothers and (b) GDM mothers, using Welch’s t-test. Bar plots on the left side display the mean proportion of each KEGG pathway while the dot plots on the right show the differences in mean proportions between the two-group using *P*-values.


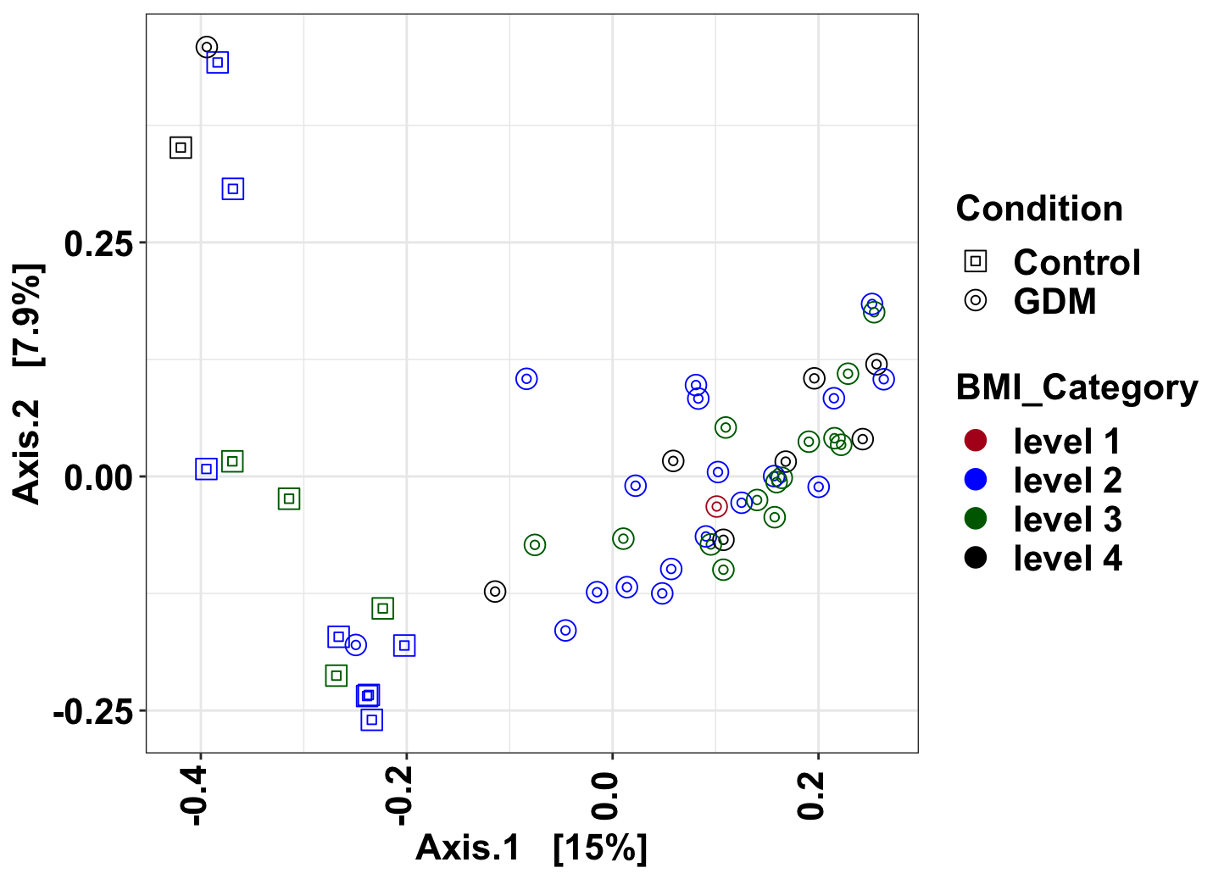


**Supplementary Figure S5.** Principal coordinate analysis plot represents the microbiome profile of mothers with different BMI levels . BMI range for each level: level-1 (below 18.5), (ii) level-2 (18.5-24.9), (iii) level-3 (25.0-29.9), and (iv) level-4 (above 30).


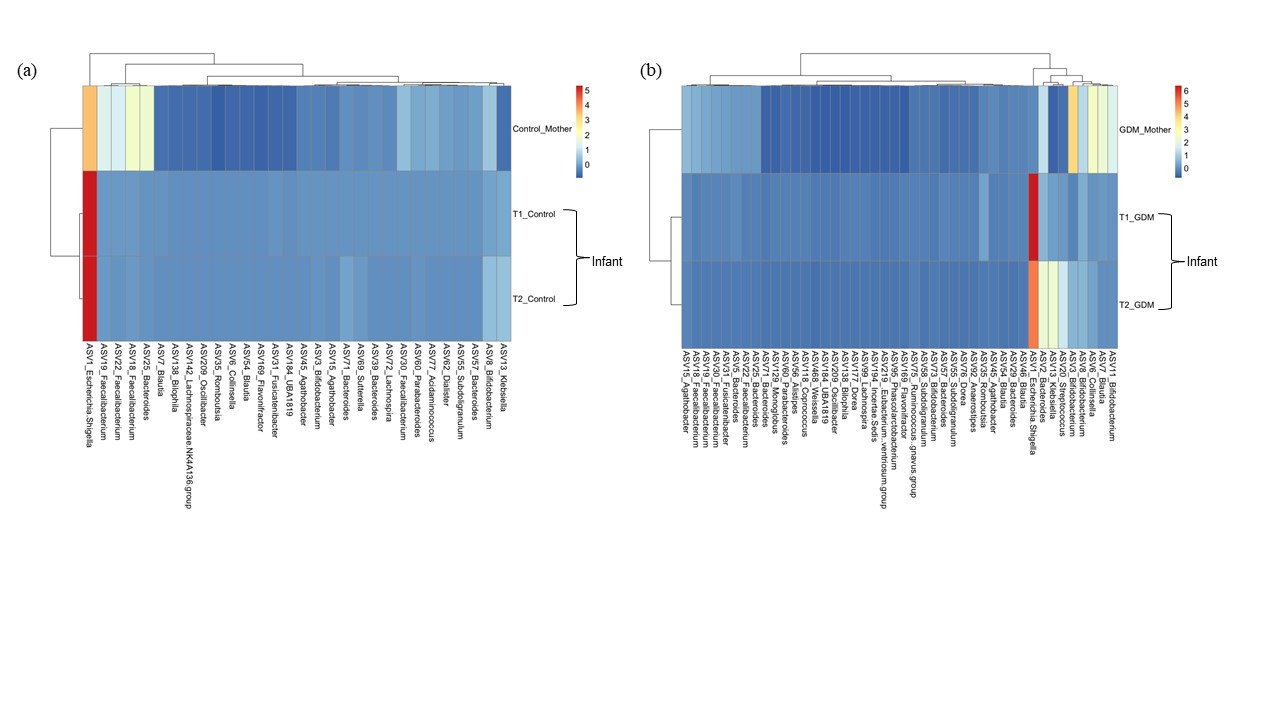


**Supplementary Figure S6.** Heatmap-based distribution of core microbiome taxa (ASVs) of mothers in their respective offspring (meconium: T1 and stool: T2). (a) Distribution of core microbial taxa in control cohort. (b) Distribution of core microbial taxa in GDM cohort. Relative abundance is scaled. Distance was calculated using Bray Curtis distance matrix; hierarchical clustering was performed Ward's method.


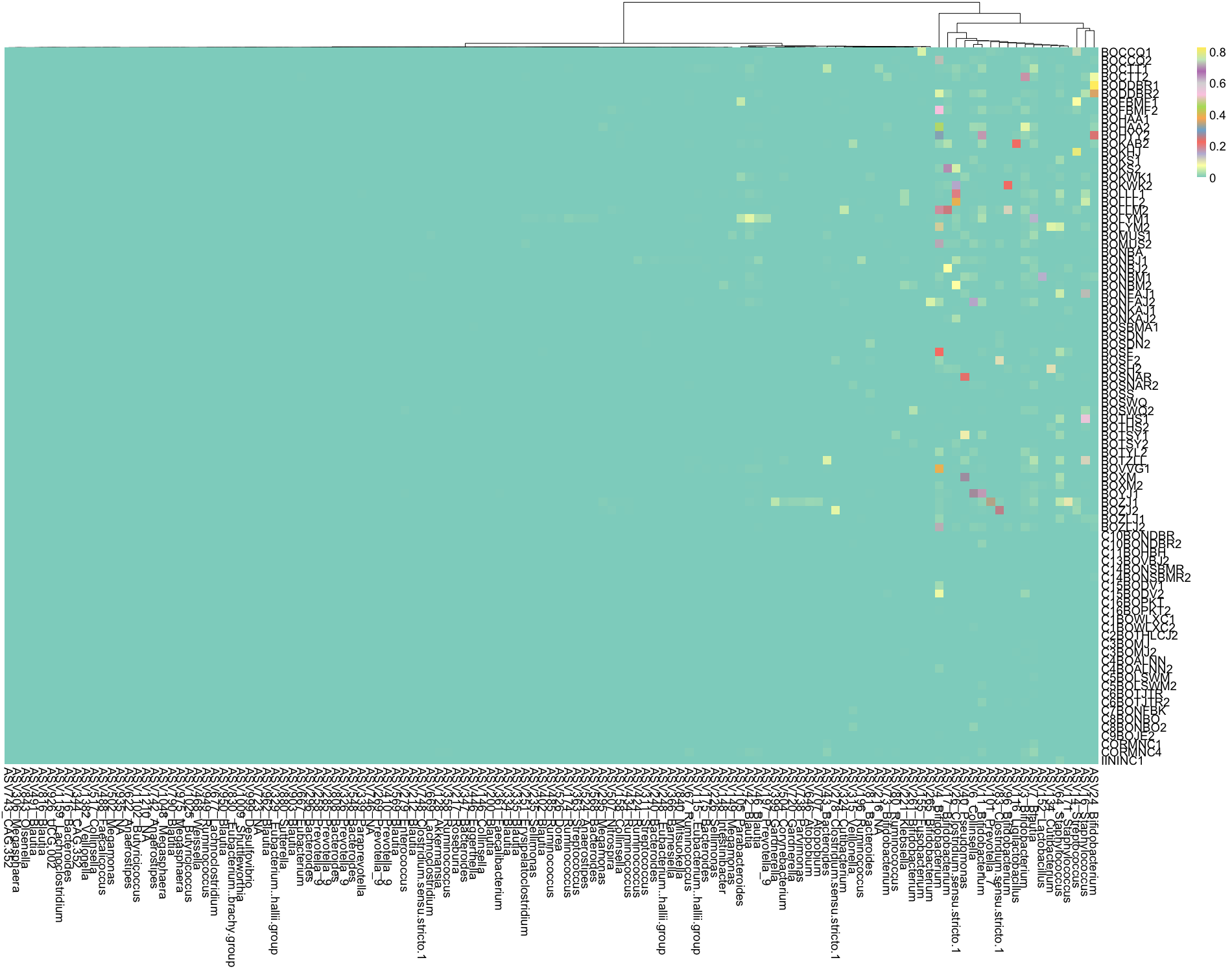


**Supplementary Figure S7.** Heatmap-based relative distribution of GDM associated microbial ASVs (log10 fold > 3.5, *P* < .001) identified (using DeSeq2) in GDM mothers in both meconium and stool samples of infants belonging to control and GDM cohorts. Hierarchical clustering was performed using Ward's method.


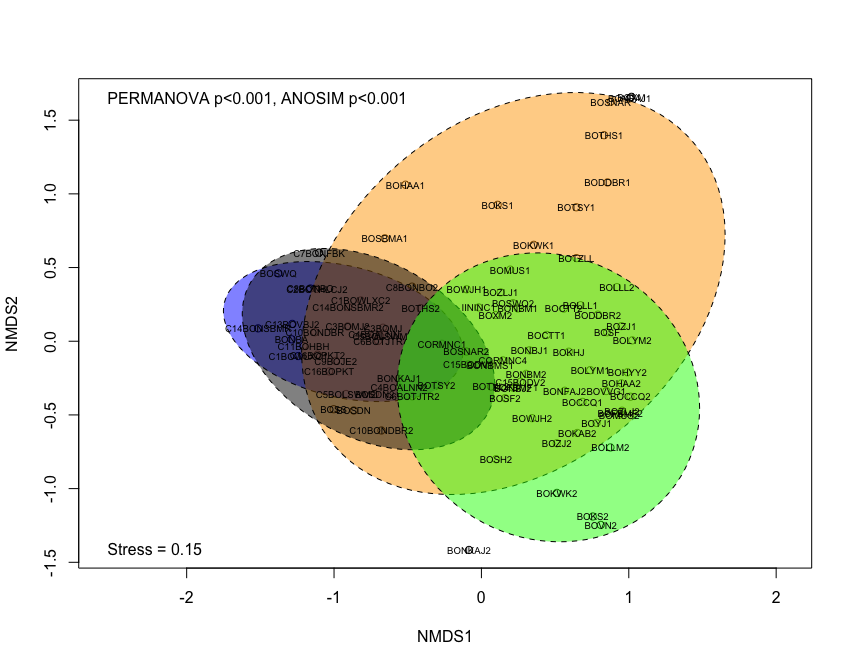


**Supplementary Figure S8.** Non-metric multidimensional scaling plot of GDM associated microbial ASVs (log10 fold > 3.5, *P* < .001) identified (using DeSeq2) in GDM mothers in both meconium and stool samples of infants belonging to control and GDM cohorts. Orange and Green: Meconium and stool samples, respectively, from infants born to GDM mothers. Blue and Grey: Meconium and stool samples, respectively, from infants born to control mothers.


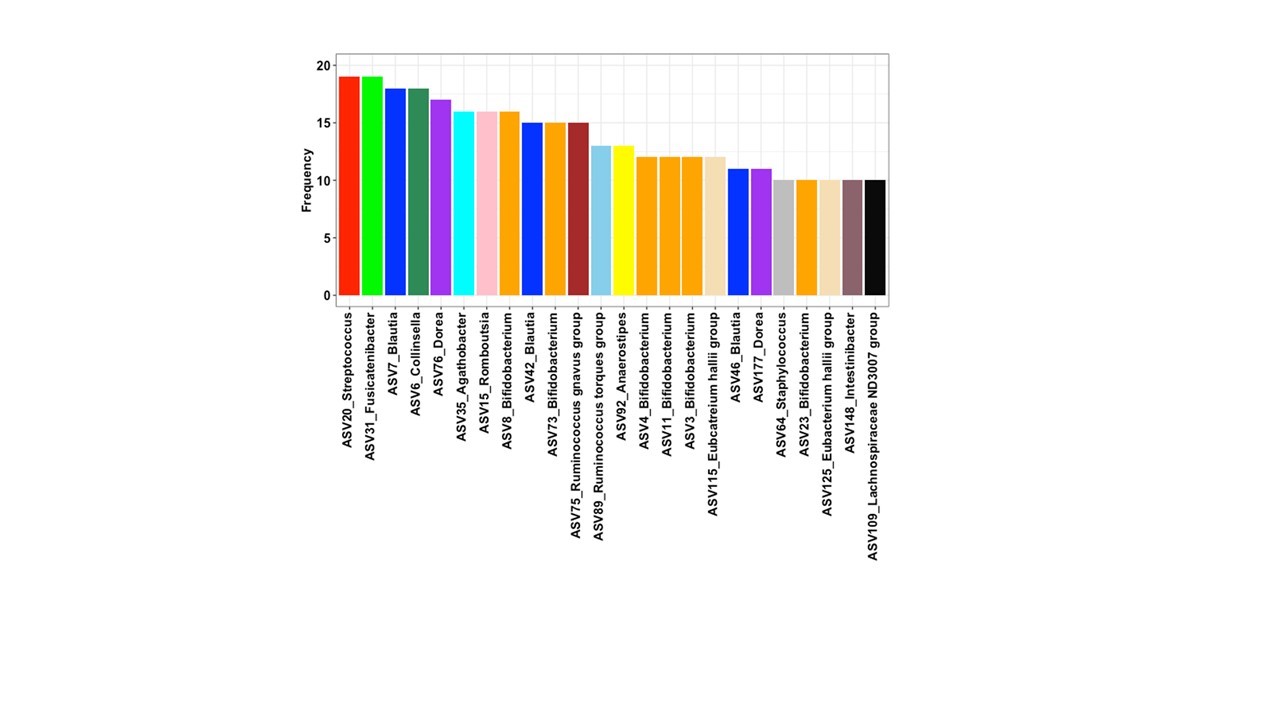


**Supplementary Figure S9.** Frequency of GDM-associated ASVs detected in mother-infant dyad pairs; the top GDM-associated AVS detected in meconium samples are presented.
